# Health status and related factors in farmers by SF-12

**DOI:** 10.1186/s40557-014-0046-8

**Published:** 2015-01-24

**Authors:** Kyungeun Park, Sooyong Roh, Jihoon Lee, Soon Chan Kwon, Mihye Jeong, Soo-jin Lee

**Affiliations:** Department of Occupational and Environmental Medicine, Hanyang University, Seoul, South Korea; Rural Development Administration, Seoul, South Korea

**Keywords:** Farmer, Health status

## Abstract

**Objectives:**

This study was performed to understand farmers’ health status by general characteristic, and to find out the related factors.

**Methods:**

All the 984 subjects were interviewed by means of a structured questionnaire and SF-12. Among them, only 812 were eligible for analysis. Statistical methods used included frequency, t-test, ANOVA, binary logistic regression with SPSS 19.0.

**Results:**

In binary logistic regression, marital status, smoking, regular exercise and monthly day off were associated with physical component score. Marital status, smoking and score of pesticide protective device wearing were associated with mental component score.

**Conclusions:**

This study suggests that effort to develop health promotion programs for workers of agricultural industry considering these results can improve their perceived health status.

## Introduction

Since the 1960s, an economic policy changed Korea from agricultural to industrial society. This economic structural change immensely affected the farmlands resulting in the exodus of young adults from rural to city becoming worse over time. It made rural areas to become an aging society. In other words, continuous shortage of labor of younger age group caused increasing labor intensity among elderly and women which are major issues today. The method of agriculture is changing from farming crops of rice in summer and barley in winter to utilizing facility horticulture like green house. But many farmers still work in a classical method of small scale intensive labor [1,2].

There are some disadvantages with respect to provision of medical care in a rural society. Excessive physical labor, increasing number of female farmers, low socioeconomic status due to lack of education, poor sanitation environment, intensive labor industry and lack of concern about health are disadvantages. Thus, farmers have difficulty utilizing health related facilities. Besides, they must endure outdoor and household work themselves even at their advancing years due to lack of manpower. As a result, farmers’ physical and mental function tend to deteriorate rapidly [3,4].

Although agriculture plays a major role in the food production industry, farming population decreased from 10,830,000 in 1980 to 3,060,000 in 2010. But it still comprises 6.3% of the nation’s population (48,580,000) which is one of the reason why farmers’ health care is important [5].

A national approach is necessary to solve farmers’ health care problem because it could affect national food supply. A planned health promotion program is needed before they are affected with a disease. In other words, farmers’ health promotion planning and action may maximize their health potential which can extend life and reduce health care cost. It can solve individual’s basic health needs and increase productivity in the agricultural industry as a result [6].

Developing and distribution of standardized program is needed for effective farmers’ health promotion. Understanding of farmers’ health status and related factors must precede the purpose.

There are various tools to evaluate self-health awareness. SF-36 which Ware et al developed is verified on reliability and validity among others. It was used on previous workers’ health status evaluations several times [7-11]. SF-12 which has 12 physical and psychological questions is a simpler tool of SF-36 for convenient use. Because 36 questions are too many to answer by elderly farmers, question response rate and accuracy may not be good. Therefore, SF-12 was utilized [12,13].

This study was conducted to evaluate farmers’ health status and related factors in order to develop farmers’ health promotion program.

## Materials and methods

### Subjects

Farmers who live in farm work safety model demonstration town were the subjects of this study. In nine provinces, 18 town 984 people agreed to and joined this study. We carried out a survey using questionnaires. The final subjects were 812 people after excluding inappropriate questionnaires.

### Survey tool

The questionnaire was composed of sociodemographic factors, life habit factors, occupational characteristics, and subjective health status (SF-12). Occupational characteristics were composed of pesticide exposure (total, annual, and daily), annual labor period, daily labor period, and monthly leave. Total pesticide exposure was divided into 3 groups, other variables were divided into 2 groups. The scores of pesticide protective device wearing and the pesticide exposure rule observance were divided into 2 groups by median value.

Subjective health status evaluation was done using SF-12. This tool has 12 questions composed of physical and mental components. The physical component is subdivided into physical functioning, role physical, bodily pain, and general health. The mental component is subdivided into mental health, role emotional, social functioning, and vitality. Each question was considered a 100 point. High score meant good health status.

### Analytical method

Collected material was analyzed by SPSS 19.0. Analysis of the frequency was used for the general characteristics of the subjects. In order to know the health status of the general characteristics, we conducted t-test and analysis of variance (ANOVA). We conducted binary logistic regression to control variables which can affect SF-12 score.

## Results

### General characteristics of subjects

Of the 812 research subjects (397 men and 415 women), the average score was 61.4, with 233 subjects (28.7%) aged between 70-79 years. Among the total, 657 (80.9%) subjects reported having a spouse at the time. In addition 227 (28.0%) subjects were smokers, 344 (42.4%) subjects were alcohol consumers, and 372 (45.8%) subjects exercised regularly (Table 1).

**Table 1:** **General characteristics of subjects (N = 812)**

**Variables**		**Number**	**%**
Gender	Male	397	48.9
	Female	415	51.1
Age(years)	≤49	137	16.9
	50-59	211	26.0
	60-69	205	25.2
	70-79	233	28.7
	≥80	26	3.2
Marital status	Single	217	19.1
	Married	657	80.9
Smoking*	NO	557	68.6
	Yes	227	28.0
Alcohol*	NO	453	55.8
	Yes	344	42.4
Regular exercise*	NO	436	53.7
	Yes	372	45.8

*excluded no-response.

### Health status (SF-12) of subjects

The average score of SF-12 was 52.66 ± 14.03. The physical component score was 52.97 ± 15.61. Physical functioning was 63.76 ± 35.17, role physical was 61.44 ± 31.99, bodily pain was 28.17 ± 11.00, and general health was 67.46 ± 10.52. The mental component score was 52.35 ± 17.19. Mental health was 72.49 ± 24.81, role emotional was 76.83 ± 20.00, social functioning was 18.78 ± 3.18, and vitality was 39.99 ± 15.04 (Table 2).

**Table 2:** **Health status(SF-12) of subjects (N = 812)**

**Scale**		**Mean ± SD**
SF-12		52.66 ± 14.03
	Physical component score	52.97 ± 15.61
	PF(Physical functioning)	63.76 ± 35.17
	RP(Role physical)	61.44 ± 31.99
	BP(Bodily pain)	28.14 ± 11.00
	GH(General health)	57.46 ± 10.52
	Mental component score	52.35 ± 17.19
	MH(Mental health)	72.49 ± 24.81
	RF(Role emotional)	76.83 ± 20.00
	SF(Social functioning)	18.78 ± 3.18
	VT(Vitality)	39.99 ± 15.04

### The health status by general characteristics

Table 3 shows health status by general characteristics. The women’s mental component score was 51.08 ± 16.77 and the mens’ score was 53.68 ± 17.55. The physical component score of the subjects who had a spouse was 54.04 ± 15.32, and the mental component score was 53.11 ± 17.11. The physical component score of the subjects who did not have a spouse was 48.42 ± 16.06, and the mental component score was 49.11 ± 17.23. The smoker’s physical component score was 57.38 ± 14.34 and the mental component score was 55.13 ± 17.52. The drinker’s physical component score was 56.04 ± 14.30 and the mental component score was 54.13 ± 16.06. The non-drinker’s physical component score was 50.51 ± 16.19 and the mental component score was 50.68 ± 17.55. The regular exercise group’s physical component score was 54.40 ± 15.56 which was higher than the non-exercise group’s score (51.67 ± 15.61). The regular exercise group’s mental component score was 53.53 ± 16.28 and the non-exercise group was 51.12 ± 17.74. The physical component score depended on the total pesticide exposure period. Post-hoc comparison result, less than 20 years exposure group and more than 36 years exposure group showed a significant difference. Less than 8 hours labor group showed a higher physical component score than exceeding 8 hours labor group. Less than 4 days monthly day off group represented a higher physical component score than exceeding 4 days monthly day off group. The group of good protective device wearing received more scores in both components significantly. Age, annual pesticide exposure, daily pesticide exposure, annual labor period and score of pesticide exposure rule observance did not make any significant difference.

**Table 3:** **The health status by general characteristics (N = 812)**

**Variables**		**Physical component score**		**Mental component score**	
		**Mean ± SD**	**p value** ^**†**^	**Mean ± SD**	**p value** ^**†**^
Gender	Male	52.50 ± 15.82	0.403	53.68 ± 17.55	0.031
	Female	53.41 ± 15.42		51.08 ± 16.77	
Age(years)	≤49	53.78 ± 15.62	0.064	53.13 ± 18.21	0.523
	50-59	52.71 ± 15.56		52.16 ± 16.83	
	60-69	53.95 ± 15.14		52.27 ± 17.79	
	70-79	50.93 ± 15.94		51.54 ± 16.64	
	≥80	61.20 ± 14.11		57.57 ± 14.69	
Marital status	Single	48.42 ± 16.06	<0.001	49.11 ± 17.23	0.009
	Married	54.04 ± 15.32		53.11 ± 17.11	
Smoking^‡^	NO	51.14 ± 15.76	<0.001	50.83 ± 16.75	0.001
	Yes	57.38 ± 14.34		55.13 ± 17.52	
Alcohol^‡^	NO	50.51 ± 16.19	<0.001	50.68 ± 17.55	0.004
	Yes	56.04 ± 14.30		54.13 ± 16.06	
Regular exercise^‡^	NO	51.67 ± 15.61	0.013	51.12 ± 17.74	0.046
	Yes	54.40 ± 15.56		53.53 ± 16.28	
Total pesticide exposure(years)^‡^	≤20	55.41 ± 14.53	0.016	54.00 ± 15.55	0.067
	21-36	53.32 ± 14.48		52.94 ± 16.16	
	>36	51.48 ± 15.79		50.54 ± 17.95	
Annual pesticide exposure(days)^‡^	≤14	53.91 ± 15.44	0.602	53.23 ± 15.54	0.340
	>14	53.31 ± 14.89		52.02 ± 17.57	
Daily pesticide exposure(hours)^‡^	≤2	52.39 ± 14.91	0.058	51.29 ± 16.75	0.070
	>2	54.59 ± 15.23		53.60 ± 16.46	
Annual labor period (months)^‡^	≤9	52.85 ± 15.81	0.141	51.84 ± 16.95	0.112
	>9	54.48 ± 14.75		53.76 ± 16.33	
Daily labor period (hours)	≤8	54.44 ± 15.15	0.005	52.75 ± 17.28	0.483
	>8	51.34 ± 15.93		51.90 ± 17.11	
Monthly day off(days)^‡^	≤4	52.06 ± 15.23	0.012	52.63 ± 16.42	0.736
	>4	54.91 ± 15.46		52.21 ± 16.71	
Score of pesticide protective device wearing	≤15	51.54 ± 16.17	0.010	50.01 ± 17.43	<0.001
	>15	54.36 ± 14.94		54.64 ± 16.66	
Score of pesticide exposure rule observance	≤20	52.66 ± 15.59	0.568	51.89 ± 17.17	0.444
	>20	53.28 ± 15.65		52.81 ± 17.23	

^†^p value by t-test or ANOVA.

^‡^excluded no-response.

### Factors related with health status

The physical and the mental component scores of SF-12 were divided into 2 groups. One was the high score group and the other was the low score group by median value. Binary logistic regression analysis was done with dependent variables which showed significant difference in univariate analysis.

On the physical component score, the odds ratio of the subjects who had a spouse was 1.89 (95% CI = 1.21-2.95), The smoker group’s odds ratio was 2.24 (95% CI = 1.56-3.21), the regular exercise group’s odds ratio was 1.37 (95% CI = 1.01-1.86), and more than 4 days monthly day off group’s odds ratio was 1.54 (95% CI = 1.11-2.14) (Table 4).

**Table 4:** **Factors related with physical component score by binary logistic regression analysis**

**Variables**		**Adjusted OR**	**95% C.I.**
Marital status	Single	1.00	
	Married	1.89	1.21-2.95
Smoking	No	1.00	
	Yes	2.24	1.56-3.21
Alcohol	No	1.00	
	Yes	1.37	0.99-1.89
Regular exercise	No	1.00	
	Yes	1.37	1.01-1.86
Total pesticide exposure(years)	≤20	1.00	
	21-36	1.01	0.65-1.51
	≥37	0.75	0.51-1.11
Daily labor period (hours)	≤8	1.00	
	>8	0.92	0.66-1.29
Monthly day off(days)	≤4	1.00	
	≥5	1.54	1.11-2.14
Score of pesticide protective device wearing	≤15	1.00	
	≥16	1.19	0.85-1.67

On the mental component score, the odds ratio of the subjects who had a spouse was 1.64 (95% CI = 1.12-2.42), The smoker group’s odds ratio was 1.57 (95% CI = 1.14-2.16), The odds ratio of the high score group of pesticide protective device wearing was 1.57 (95% CI = 1.17-2.10) (Table 5).

**Table 5:** **Factors related with mental component score by binary logistic regression analysis**

**Variables**		**Adjusted OR**	**95% C.I.**
Gender	Male	1.00	
	Female	0.85	0.64-1.14
Marital status	Single	1.00	
	Married	1.64	1.12-2.42
Smoking	No	1.00	
	Yes	1.57	1.14-2.16
Alcohol	No	1.00	
	Yes	1.26	0.93-1.72
Regular exercise	No	1.00	
	Yes	1.17	0.87-1.57
Score of pesticide protective device wearing	≤15	1.00	
	≥16	1.57	1.17-2.10

## Discussion

Korea’s agriculture plays a major role in the food production industry and concern for farmer’s health is increasing. Especially, farmers tend to be more elderly compared to any other industry. Understanding farmers’ health status is basically an important step. In this study, marriage status, smoking, regular exercise, monthly day off and pesticide protective device wearing were significant variables in farmers’ health status.

SF-12 score of the subjects was 52.66 out of 100. The study of Cha BS et al (1998) which showed the assessment of workers’ health status by SF-36 showed 69.61, manufacturer male employees’ assessment by SF-36 (Kim SA et al, 2006) was 78.44, and Lee SM (2010)’s study of large workplace employees in Daejeoun and Chungchung health status assessment by SF-12 showed 75.75 [11,14,15]. All of the above studies showed higher scores than this study. It may be because the farmers were older or there were more number of females or had less education or lower economic level than in the other workplace. In Jun JY’s study which evaluated elderly in a rural area by SF-36 revealed 56.15. It was higher than this study. Such study included subjects who were all elderly in an area regardless of farming. But it was difficult to compare because the number of subjects was too small [16].

On the mental component score, the females’ score was lower than the males’. It corresponded with previous studies which showed that the females generally had a lower health status than males [12,15,17-19]. There are some points to be considered. Nettleton (1995) explained that women work double hours at home and at work which causes negative effect on health. On the other hand, MacIntyre (1993) said that women tend to know more about their health status, and men exaggerate their health [20,21].

This study did not show significant difference in health status according to age. It does not follow previous studies which explains that health status decreases with age [12,17,18,22-24]. But some studies in elderly subjects showed that health status does not have correlation with age [25]. And the average age of the subjects was 64.1 which was high and many of them were more than their 60’s in this study. Therefore, it may not appropriate to compare.

Health status in married group was higher than in single group. Previous studies showed similar results. Existing spouse is helpful in physical health management and psychological stability [14,16,26,27].

Smokers’ health status score was higher than nonsmokers’ score. It was similar to previous studies [28,29]. However, there are many reports which explain that smoking has negative health effect and stop smoking in old age is helpful in improvement of health and quality of life [30]. And smoking can be a confounding factor. Alcohol did not have any significant correlation. The regular exercise group had a higher health status score. It corresponded with other studies [28,31,32].

Pesticide exposure did not show any significant relation. Long time pesticide exposure group tended to have low scores, but was not significant after revision. Meanwhile pesticide protective device wearing had a positive effect, especially the mental component. People who made efforts to wear protective device tended to have more concern about health. It was meaningful that there were few previous studies concerning the association of protective device and health. Longer monthly day off group had higher physical component scores. There were some similar results about the association between working day and health [33-35].

There are some limitations in this study. First of all, this study was carried out targeting 9 provinces in the country, but the sampling count per each town was too small. Therefore, it cannot be generalized among all farmers. And there were many differences in working conditions by crop. A close investigation was needed further. Secondly, this study was a cross-sectional research. The association of variables was found to exist, but the order of time was not clear. Lastly, there were omitted variable bias. The subjects were old age, but a questionnaire was used. BMI, income level, education level and sleeping hours which are related to health were omitted [25,36].

## Conclusion

Farmers had disadvantages in medical approach. Developing a program is needed to manage them. According to this study, life style improvement, education of pesticide use like protective device wearing and proper working time and rest have to be considered.

Further research on the subjects after application of the improvement program based on this study is necessary.
